# Artificial Neural Networks for Discrimination of Automotive Clear Coats by Vehicle Manufacturer

**DOI:** 10.3390/s26072260

**Published:** 2026-04-06

**Authors:** Barry K. Lavine, Collin G. White, Douglas R. Heisterkamp

**Affiliations:** 1 Department of Chemistry, Oklahoma State University, Stillwater, OK 74078, USA; collin.white@okstate.edu; 2 Department of Computer Science, Oklahoma State University, Stillwater, OK 74078, USA; douglas.r.heisterkamp@okstate.edu

**Keywords:** neural networks, multilayer perceptrons, automotive clear coats, deep learning, pattern recognition, forensic automotive paint analysis, spectral preprocessing

## Abstract

Modern automotive paints have a thin undercoat and color coat layer protected by a thick clear coat layer. All too often, only the clear coat layer of the automotive paint is recovered at the crime scene of a vehicle-related fatality. Searches for motor vehicle paint databases of clear coats using commercial software typically generate large hitlists that are difficult for a forensic paint examiner to work through unless additional information is provided for the search. To address this problem, deep learning has been applied to the infrared spectra of automotive clear coats to identify patterns in their spectra indicative of the motor vehicle manufacturer. An in-house automotive paint library of 2796 clear coat infrared spectra from six automotive manufacturers and 100 assembly plants was partitioned into training, validation, and prediction sets. Each spectrum has 1880 measurements over the spectral range of 4000 cm^−1^ to 376 cm^−1^. Several multilayer perceptron neural network models, each with three hidden layers, were developed that achieved high classification success rates for the training, validation, and prediction sets. The addition of convolutional layers to the deep learning neural network models did not improve the performance of these models.

## 1. Introduction

Motor vehicle paint is a multilayered coating that protects a vehicle from corrosion while providing it with the desired finish. Motor vehicle paint [1] typically consists of four layers: clear coat, color coat, surfacer primer, and electro-coat. All layers in an original equipment manufacturer (OEM) automotive paint have a specific purpose, either to protect the substrate from corrosion and chipping or to provide a finish with specific aesthetic features. All too often, only the clear coat layer of an automotive paint is recovered at the crime scene of a vehicle-related fatality, such as a hit and run, where injury or death to a pedestrian has occurred. As most clear coats are not colored and do not contain inorganic fillers or extenders with which to further differentiate one clear coat from another, OEM clear coats typically cannot be differentiated in a motor vehicle paint database search. The similarity of the FTIR spectra of automotive clear coats also results in poor discrimination using standard IR spectral search algorithms that are programmed into the data systems of most FTIR spectrometers. Without additional information (e.g., the IR spectra of other paint layers or the identity of the motor vehicle manufacturer), the hitlist generated from the search of an automotive paint database, such as paint data query (PDQ), would be so large that it would be difficult for the forensic paint examiner to effectively narrow down the samples in the list.

At present, the identity of the motor vehicle manufacturer cannot be inferred from the IR spectrum of an automotive clear coat. This study demonstrates that the motor vehicle manufacturer (e.g., Toyota, Nissan, and Honda) of an unknown OEM automotive clear coat can be determined from its mid-IR spectrum using ANN techniques after judicious standardization and spectral data preprocessing, which is the novelty and the innovation highlighted in this study. Information about the motor vehicle manufacturer is crucial in the forensic examination of automotive clear coats, as it can help to quantify the general discrimination power of OEM automotive clear coat comparisons encountered in actual case work and further efforts to succinctly communicate the significance of the evidence to the courts.

A variety of machine learning techniques have been applied to FTIR spectral data in recent years, including multilayer perceptrons [2], convolutional neural networks [3], support vector machines [4], random forest [5], principal component analysis [6], and K-NN [6]. Buydens and coworkers [7] demonstrated that a convolutional neural network can outperform established machine learning classification algorithms for several infrared, Raman, and near-infrared datasets where each sample is represented by a spectrum and the classes are different geographic areas of origin or sample type (e.g., strawberry versus non-strawberry juices). For the FTIR spectra of automotive clear coats, principal component analysis is the method of choice [8]. However, FTIR spectra of automotive clear coats cannot be differentiated based on the motor vehicle manufacturer (e.g., Toyota, General Motors, Ford, etc.) using machine learning techniques. Recently, Lavine and Sandercock [9,10,11] have shown that pattern recognition techniques can improve the quality of an automotive clear coat library search by decreasing the size of an automotive paint library for a specific vehicle manufacturer to an assembly plant or assembly plants using wavelets [12] to preprocess the FTIR spectral data and a genetic algorithm for pattern recognition [13] to identify wavelet coefficients that optimize separation of the spectra characteristic of the assembly plant of the vehicle from which the paint sample originated in a plot of the two or three largest principal components of the data. To apply this pattern recognition approach to actual casework, the forensic automotive paint examiner must determine the motor vehicle manufacturer of the unknown automotive clear coat. Currently, the motor vehicle manufacturer of an automotive clear coat cannot be determined by examining its IR spectrum using library search algorithms or pattern recognition techniques.

To address this problem, a multilayer perceptron (MLP) neural network equipped with three hidden layers and utilizing deep learning [14,15] has been applied to the FTIR spectra of OEM automotive clear coats to enhance their discrimination power for forensic automotive paint comparisons. Specifically, we seek to identify the motor vehicle manufacturer (e.g., Toyota, Nissan, or Honda) from the IR spectrum of an unknown automotive clear coat, as this information may assist in the forensic examination of OEM clear coats. For this study, an “in-house” spectral library of 2796 mid-IR spectra of OEM automotive clear coats from six automotive manufacturers and 100 assembly plants obtained from the Royal Canadian Mounted Police was partitioned into a training set of 2237 spectra and an external prediction set of 559 spectra. Each IR spectrum after standardization consisted of 1880 points over the spectral range of 4000 cm^−1^ to 376 cm^−1^. After spectral baseline correction and removal of sample outliers, the performance of the MLP models developed from the training set data was evaluated using the technique of ten-fold cross-validation. Several MLP neural network models, each consisting of an input layer, three hidden layers, and an output layer, were developed that achieved high classification success rates for similar spectra in both the training set and external prediction set. Standardization of the spectra, followed by data preprocessing (baseline correction and outlier removal), was crucial for achieving these high classification success rates.

## 2. Materials and Methods

A pattern recognition approach was utilized to solve the class membership problem highlighted in this study. A major requirement for a successful pattern recognition study is a well-designed dataset. The classes must be carefully selected with respect to the problem, and the samples in each class should in some way be similar. Furthermore, the data measured on the samples should be related to this similarity. The discrimination of automotive clear coats by vehicle manufacturers using mid-IR spectroscopy meets these requirements. The in-house spectral library database is well designed, as every assembly plant in North America between 2000 and 2010 is well represented, and the six automotive manufacturers (General Motors, Ford, Chrysler, Honda, Toyota, and Nissan) comprising the six classes account for 86% of the vehicles sold in North America [16]. Each automotive clear coat is represented by a mid-IR spectrum, and the IR spectrum (in turn) is related to the chemical composition of the automotive clear coat, which can be directly correlated to the identity of the motor vehicle manufacturer. In addition, FTIR spectra were standardized to ensure that each IR spectrum was represented by the same number of points, and the data were then baseline corrected to reduce background and noise. Finally, outlier analysis was performed on the baseline-corrected spectra to identify and remove discordant observations that otherwise would adversely affect the performance of the pattern recognition method used for classification.

In this study, a multilayer perceptron (MLP) with four trainable dense layers (of which three were hidden layers) served as the discriminant, with each node of the MLP receiving inputs from the previous layer and transferring its output to the nodes in the next layer. The construction of the class boundaries in the hidden layers was evaluated in the output layer. The input layer, which is not trainable, served only to store the spectral data of the automotive clear coats. The MLP was trained using the entire spectrum normalized to unit length (i.e., L2 normalization) or a segment of the spectrum normalized to unit length. The term deep learning is used in this study since neural networks that integrate two or more hidden layers are called deep neural networks, and the practice of using deep neural nets is referred to as deep learning.

### 2.1. Automotive Paint Dataset

The 2796 transmission OEM automotive clear coat IR spectra (see Table 1) comprising an in-house automotive paint library that spans six automotive manufacturers was provided by the RCMP Forensic Laboratory (Edmonton, AB, Canada) and was obtained from street samples and factory panels. These six automotive manufacturers represent 86% of vehicles sold in North America in 2015. Four different FTIR spectrometers were used to collect clear coat IR spectra: Bio-Rad 40A (Hercules, CA, USA), Bio-Rad 60A (Hercules, CA, USA), and two Thermo Nicolet 6700 (Waltham, MA, USA). Each spectrometer was equipped with a DTGS detector and run at 4 cm^−1^ resolution. All clear coats analyzed were between 3 and 4 micrograms in mass and were seated in a high-pressure diamond transmission diamond anvil cell [17,18] mounted in a Harrick 4x or a Harrick 6x beam-condenser to push more of the IR beam through the sample and enhance the signal. All clear coat samples have approximately the same thickness because the spectra were collected under the same conditions and in such a way that the largest absorbance peak (around 1730 cm^−1^) in all 2796 OEM clear coat FTIR spectra had a percent transmittance near 10%. Although the pressure applied by the high-pressure diamond cell significantly reduced the thickness of the original clear coat layer, fringing was not observed in any of the spectra used in this study.

**Figure 1:**
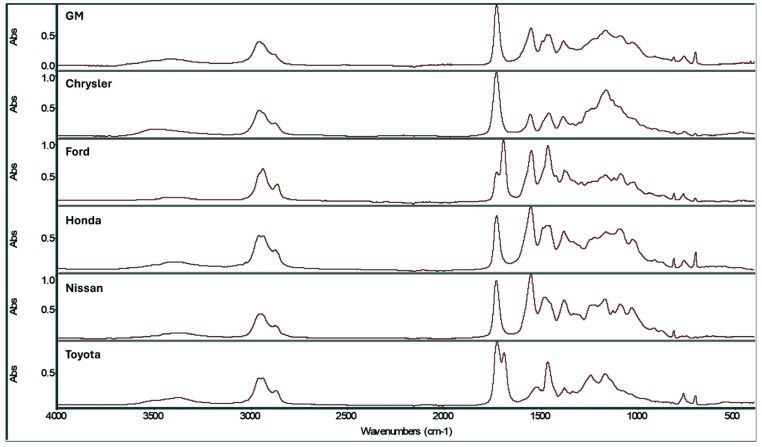
Representative spectra from the six automotive manufacturers.

The number of points per spectrum collected in the wavenumber range of 4000 to 376 cm^−1^ by the two Bio-Rad FTIR spectrometers (1944 points) differed from the number of points collected by the two Thermo-Nicolet spectrometers (1878 to 1948 points) at the same resolution and same spectral range. Band shifting was also observed in spectra collected on all four instruments. These problems were resolved by helium–neon laser frequency normalization [19] using OMNIC (Thermo-Nicolet) software as an editor to process the Bio-Rad spectra and the spectra from the two Thermo-Nicolet instruments. Each spectrum was normalized to the helium–neon laser frequency of 15,798.0 cm^−1^, which corresponded to the frequency at the aperture setting, making the sample peak positions independent of the aperture setting. After frequency normalization, the FTIR spectra collected on the two Bio-Rad instruments were comparable to the clear coat spectra collected on the two Thermo-Nicolet instruments. To validate proper spectral alignment, FTIR spectra of known samples measured on both the Bio-Rad and Thermo-Nicolet instruments were compared using vector subtraction before and after performing frequency normalization. Subtraction yielded a nonzero response at each wavelength before frequency normalization but zero at each point after normalization, indicating that spectral alignment had been achieved. After frequency normalization, all FTIR spectra (4000 to 376 cm^−1^) were represented by 1880 points. Figure 1 shows frequency-normalized clear coat IR spectra representative of the six automotive manufacturers (General Motors, Chrysler, Ford, Honda, Nissan, Toyota).

### 2.2. Data Preprocessing

All OEM clear coat IR spectra in the in-house automotive paint database were baseline corrected using the rubber band method [20] that was first implemented in MATLAB (version R2024A, Natick, MA, USA) and later in Python. This method was determined to be the most effective for reducing the background from OEM automotive clear coat IR spectra. For outlier analysis, the generalized distance test [21] was applied to each class (i.e., vehicle manufacturer) in the training set (see Table 2) to identify and delete anomalous samples. The generalized distance test was performed using the program SCOUT [22,23] run on a Dell Dimension 8300 personal computer under Windows XP. Each FTIR spectrum was also normalized to unit length, which standardized the optical path length of each OEM clear coat sample. Baseline correction, outlier analysis, and vector normalization were crucial for the development of the deep learning models highlighted in this study.

### 2.3. Deep Learning

Deep learning was performed using an ASL Marquis C739-T computer with two Intel Xenon Gold 6348 processors (each having 28 dual-thread cores) and two EVGA GeForce RTX 3080Ti FTW3 graphics cards (10240 Cuda cores each). The computer has a total of 11 TB of hard drive space, sufficient to store the metadata produced by a deep learning run. Google’s Tensor flow with the Keras (version Keras 3.4.1) application program interface was used to develop neural network models for this study.

OEM clear coat IR spectra from the in-house library were classified using a multilayer perceptron (MLP) designed to process IR spectral data as input and assign each clear coat to one of six classes. The MLP developed with the Keras toolset consisted of an input layer (to store the spectral data for standardization and processing), three hidden layers (to process the information), and an output layer (to provide the results). The number of neurons in the input layer was 1880, corresponding to the number of points in the wavenumber range 4000 to 376 cm^−1^ after standardization by normalizing the helium–neon laser frequency to the same value. The MLP was trained using the full spectrum, normalized to unit length, or a segment of the entire spectrum, normalized to unit length. For each segment (1500 to 600 cm^−1^, 1844 to 667 cm^−1^, 1641 to 667 cm^−1^, 1641 to 860 cm^−1^), the spectrum of 1880 points was cropped from 1880 points to 468, 548, 506, or 406 points to obtain the desired spectral range. Gaussian noise was added to the data prior to training to ensure that spurious correlations did not confound the neural network while it trained. The expectation is that Gaussian noise would be smaller than the increment between one valid measurement and the next.

Three hidden layers were implemented, with the first containing 1000 nodes, the second 500, and the third 100. Each hidden layer employed the ReLU activation function [24] instead of a sigmoid or hyperbolic tangent function to map each node’s output as it improved training, allowed small negative gradients, and helped to reduce the gradient vanishing problem. To prevent overfitting, which was of concern due to the small training sample size, a dropout layer was included after each hidden layer, with a dropout rate of 0.35. Dropout [25] randomly inactivated a fraction of the neurons during each epoch as defined by the dropout rate, so that the MLP would not rely on a small subset of neurons to predict. Batch normalization [26] was also included with each hidden layer to prevent overfitting. The activation output from the previous layer was transformed into a standard normal distribution by subtracting the mean value from the output and dividing the output by its standard deviation. An added advantage of batch normalization is that it ensures extreme values in the previous layers do not cause vanishing gradients in the next layer, allowing each layer to learn independently of the other, as a large value in one layer does not excessively influence the calculation in the next layer and enables faster training, as there are no extreme values in the normalized outputs. As for the output layer, it was implemented using six neurons corresponding to each of the six vehicle manufacturers investigated in this study, and the layer employed a SoftMax [27] activation function. Although the SoftMax activation function is most often used as the output of a classifier to represent the probability distribution for a sample over different classes, these output probabilities should be viewed skeptically, and we chose to use the values from the output layer to make a hard prediction. Finally, the parameters of the MLP were optimized through back propagation [28] and trained using the Adam optimizer [29] with the training monitored by cross-entropy [30] as the cost function. The stopping condition used for training was the number of epochs as specified by the user.

For deep learning, the 2796 mid-IR clear coat spectra were partitioned into a training set of 2237 spectra (see Table 2) and an external prediction set of 559 spectra (see Table 3). Clear coat IR spectra comprising the prediction set were randomly selected. For cross-validation, the training set was partitioned into ten sets of approximately the same size. To evaluate the performance of the neural network models and set the meta parameters of the neural network, ten-fold cross-validation was performed on the set of 2237 spectra using two separate validation sets. A “fold” consists of combining eight partitions into a training set using one partition as the first validation set to compute the meta parameters, and the remaining fold as the second validation set, which serves as a test set. A model is trained on eight partitions with the ninth partition serving as a validation set (to select meta parameters for the model and to decide when to stop training). The tenth partition is used as a test set to evaluate the model selected by the first validation set. This process is repeated ten times, each time using a different fold as a validation set or test set. Thus, ten models are created and trained independently, each using a different partition for evaluation. Furthermore, each of the 2237 spectra is present in only one of the ten partitions for evaluations. The results from each test set are compiled to produce a robust estimate for the performance of the ten models, which is reported in this study as the prediction accuracy. Although ten models have been created by ten-fold cross-validation, only the percentage of the total number of samples correctly predicted in the ten test sets by these ten models is reported as an indication of the prediction accuracy.

The set of data—2796 FTIR spectra of 1880 points each—was transferred via a flash drive from a Dell OptiPlex (Windows 11 operating system) personal computer to an ASL Marquis C739-T computer, where it was entered into a repository as Tensor-Flow data-streams for the construction of Keras models to discriminate the OEM clear coat FTIR spectra by vehicle manufacturer (General Motors, Chrysler, Ford, Honda, Nissan, Toyota).

Keras models that were developed in this study and that possessed the necessary accuracy for forensic paint analysis are currently housed in a repository (AutomotivePaint) at www.box.com and are available upon request from the authors. The repository contains a fully functioning Windows executable that includes a Python implementation of the rubber band baseline correction algorithm that allows the user to stack the baseline with the original unknown spectrum and send it to the desired Keras model for prediction.

## 3. Results

When attempting to differentiate similar OEM clear coat FTIR spectra, baseline correction is necessary to reduce noise and ensure standardization of the FTIR spectra for deep learning. Baseline correction is also crucial for identifying outliers. This was shown in a previously published study on the discrimination of automotive clear coats by way of an assembly plant using IR and Raman spectroscopy [31]. For baseline correction, a single iteration of the rubber band was applied to each IR spectrum. However, a full iteration of the rubber band, when applied to clear coat FTIR spectra, decreased the predicted accuracy of the neural network model compared to the results obtained for the corresponding neural network model without baseline correction of the clear coat IR spectra. We attributed this to an overcorrection. Therefore, each baseline-corrected spectrum (a single iteration of the rubber band) was subtracted from the original spectrum to yield a snapshot of the background for each of the 2237 IR spectra comprising the training set. For each spectrum, the snapshot of the background was then multiplied by 0.1, 0.2, 0.3, 0.4, 05, 0.6, 0.7, 0.8, and 0.9 and then subtracted from the original spectrum to yield baseline-corrected IR spectra for the 2237 training set spectra. The value of 0.5 was selected, as it yielded the highest classification success rate for the test partition data, as determined by the MLP neural network model developed from the training set using 5000 epochs and ten-fold cross-validation. Therefore, multiplying each snapshot by 0.5 addressed the problem of baseline correction and yielded the best results for baseline correction of OEM automotive clear coat IR spectra.

As outliers will prevent a model from generalizing, outlier analysis was also performed on each class in the training set after baseline correction using the generalized distance test for the full spectral range and the four spectral regions used for model development (see Table 4). For example, 56 clear coats in the training set that encompassed the full spectral range were identified as outliers: two General Motors, 18 Chrysler, three Ford, three Honda, 14 Nissan, and 16 Toyota clear coat samples. These 56 samples were removed from the training set for studies involving the full spectral range. In all likelihood, the source of these outliers can be attributed to the formulation used to prepare these clear coats, which may have differed from those of the other clear coats for specific assembly plants of a motor vehicle manufacturer. It is not uncommon for changes to occur in the formulation used to prepare the clear coat layer of an automotive paint during a production year.

Table 5 summarizes the initial results using the test prediction folds for a set of neural network models, each consisting of four dense layers, developed from the 1880 measurements to assess the prediction accuracy of the models. Each artificial neural network (ANN) model yielded 100% correct classification for the training data. What matters, however, is obtaining good generalization using information beyond fitting the training data for class prediction. For the test partition data, as the number of epochs increased, so did the accuracy of the model. Only 500 epochs were required to obtain a classification success rate of 81.7% for the test partition data. The high prediction accuracy, even at low epoch counts, can be attributed to the ease of classifying the automotive clear coats from the interior of each class. However, more epochs were needed to classify those samples on the boundaries between classes, where the decision is more difficult to make. The 500 and 5000 runs were done without a parameter search, whereas the 50,000 runs were performed using a single sequential parameter search.

The addition of three convolutional layers to ANN4 did not improve the performance of the neural network to discriminate automotive clear coats by vehicle manufacturer. For “chemical fingerprinting” problems of the type that we are considering, the location or wavenumber of the spectral feature is important. By comparison, for images and in-text analysis, where convolutional neural networks (CNNs) have been utilized [32], it is the relative location of the features that is important, not their specific location. Although CNNs are designed for translation-invariant sets of local features, this has not provided us with an advantage for this spectral analysis. Convolutional features have a limited spatial extent that expands with multiple CNN layers, but they are also comingled with creating higher-order features. Clearly, the shape of the absorption band does not seem as important as its location in the spectrum (i.e., wavenumber). CNNs were also more sensitive to meta-parameters when trained on our data. For this reason, more complicated stopping conditions were required, such as the weighted gap between the training and first validation set, to decide when to stop training and use the model for testing. By comparison, the use of batch normalization and dropout for ANNs allowed for a simpler stopping condition, such as a fixed number of epochs.

Table 6 shows the results of a more detailed study where four spectral regions (fingerprint region) were investigated, as well as the full spectral range by the ANN-4 models. Performance is presented as the prediction accuracy (i.e., the percentage of the total number of samples correctly predicted for the motor vehicle manufacturer) using the ten test folds from the ten-fold cross-validation. The neural network models for the four spectral ranges were developed using the same number of iterations and were at the same stage of their parameter search as the dense four-layer neural network model (80,000 iterations/one parameter at a time—one pass through all parameters) developed using the full spectral range. Narrowing the spectra range from 4000 to 376 cm^−1^ to the fingerprint region increased the prediction accuracy, which was expected. It is well known that compounds with similar structures often exhibit spectral differences in the fingerprint region, as this region (unlike the region above 2000 cm^−1^) is sensitive to small changes in the chemical composition of the sample. In the case of automotive clear coats, the fingerprint region will be sensitive to differences in the chemical composition of the formulation used to prepare these polymers.

As the confusion matrix of each neural network model was similar, we chose to focus our attention on the confusion matrix for the full spectrum model to better understand the performance of the ANN4 models. Table 7 shows the confusion matrix developed using the test folds from the ten-fold cross-validation of the full-spectrum model. Prediction accuracy based on the classification success rate for the vehicle manufacturer is higher for Honda, Nissan, and Toyota than for General Motors, Ford, and Chrysler. Ford posed the greatest challenge for the classification of General Motors and Chrysler automotive clear coats, whereas General Motors and Chrysler automotive clear coats were more often mistaken for Ford than for the other three vehicle manufacturers. As for the three Japanese manufacturers, Honda is more likely to be confused with Nissan than with the other vehicle manufacturers, and Nissan is more likely to be confused with General Motors and Ford. Toyota is more likely to be confused with General Motors and Honda. From an examination of the confusion matrix, discriminating General Motors and Chrysler OEM automotive clear coats from Ford OEM clear coats was a major challenge in this study. The lower classification success rates obtained for the test folds by General Motors, Ford, and Chrysler clear coats in ten-fold cross-validation can probably be attributed to the larger number of assembly plants associated with these three vehicle manufacturers (see Table 1). Since each assembly plant uses a different formulation for the preparation of the clear coat layer, sample variability would be expected to be greater for General Motors, Chrysler, and Ford automotive clear coats. Hence, these three vehicle manufacturers would be more difficult to discriminate by FTIR spectroscopy.

Using the ten models developed from the ten-fold cross-validation for each spectral range (see Table 6), predictions from each of the ten models were made for an ensemble prediction of each sample in the external prediction set (see Table 3). In other words, for each sample in the external prediction set, the network output for each of the ten models was converted to a 0/1 prediction with the class assignment of the sample (i.e., vehicle manufacturer) determined by a majority vote. The results of these ensemble predictions are summarized in Table 8. Prediction accuracy, which is comparable to recall, is the metric most often used in the forensic analysis of automotive paint. Accuracy can be defined as the fraction of all classifications that are correct, whereas precision is the ability of the classifier not to classify a negative sample as positive, and recall is the ability of the classifier to identify all positive samples. The F1 score is a geometric average of the precision and recall scores. Using this set of metrics instead of only prediction accuracy allows the reader to better understand the differences in performance between these discriminants. From Table 8, it is evident that the Keras model developed for the spectral range of 1641 to 667 cm^−1^ yielded the best results: 89.98% for the ten test folds from the ten-fold cross-validation and 88.3% for the ensemble predictions of the 559 spectra comprising the external test set.

Table 9 shows the confusion matrix for the ensemble predictions of the 559 spectra comprising the external prediction set using the ten neural network models developed for the spectral range 1641 to 667 cm^−1^. Prediction accuracy was highest for Toyota and lowest for General Motors. As in the case of ten-fold cross-validation, General Motors and Chrysler automotive clear coats were more often mistaken for Ford than for the other three vehicle manufacturers.

For all MLP models, the prediction accuracy using the external test set is comparable to the ten-fold cross-validation accuracy, suggesting that overfitting of the data has not occurred when developing each of these ANN dense four-layer neural network models. These results constitute direct evidence that mid-IR spectra of OEM clear coats contain information about the vehicle manufacturer.

## 4. Discussion

This study has addressed critical knowledge currently lacking in the development and implementation of neural network models to differentiate similar FTIR spectra. Small improvements in spectral preprocessing techniques, such as baseline correction and normalization of each FTIR spectrum to unit length, have been shown to have a marked difference in classifying similar spectra into their respective class (one of six motor vehicle manufacturers), and our result lies in agreement with the opinion expressed by other workers in the field [33]. Although statistical differences may exist between spectra from different classes, their chemical composition may be identical. Conversely, spectra from each of these classes may be comparable based on comparison statistics, but their composition may be different. The problem herein lies with the neural network not discriminating chemical differences—only first-order shape differences that may be due to instrument measurement differences. The issues that are causing spectroscopic differences for samples with similar composition include bandpass and resolution settings, the type of mathematical corrections (e.g., apodization) used for interferogram collection, and instrument settings at the time the samples are measured. All these factors are convolved into the spectrum, and instrumental differences cannot be deconvolved from differences in chemical composition for spectral discrimination. Further compounding this problem is overfitting. Therefore, a strategy to minimize overfitting was adopted in this study, which included adding Gaussian noise to the data prior to analysis and utilizing both batch normalization and dropout layers with each hidden layer of the MLP.

Conventional wisdom in chemometrics, which has advocated modeling of all the data collected, has often been at odds with the view from statistics [34], that a carefully selected set of variables, resulting in a “thin” training set matrix, usually makes a better model. However, simple selection of variables using the net analyte signal is not advisable [35], and getting a useful, thin data matrix that is stable to changes in the instrument over time remains a significant challenge. Many authors have tried to use stepwise variable selection either prior to or as part of a method, such as PLS [36,37], but stepwise methods only work with a small number of variables, while the typical spectral response has thousands of variables, so these approaches are not useful for selecting wavelengths other than eigen-vector-based variables in chemical responses [38].

The results from this study should also be considered in the context of a previously published study by Lavine and coworkers [39] who demonstrated that pattern recognition methods when applied to the fingerprint region (1641–667 cm^−1^) of the IR spectra of the two undercoat layers and clear coat layer for the same six-way classification problem could identify the motor vehicle manufacturer of an OEM paint. A hierarchical discriminant was developed using 1596 OEM paint samples obtained from PDQ. The discriminant was successfully validated (with 100% prediction accuracy) using an external prediction set of 183 OEM paint samples also obtained from PDQ. When comparing the training set and prediction set results using only automotive clear coats to this earlier study that utilized all three layers, one can infer that most of the discriminatory information about the vehicle manufacturer is captured by the clear coat layer. Although automotive clear coats cannot be differentiated in motor vehicle paint database library searches due to the similarity of their spectra, judicious application of spectral preprocessing techniques, such as baseline correction and normalization of each spectrum to unit length, when coupled to a deep neural network configured to minimize overfitting can successfully extract information about the motor vehicle manufacturer from the IR spectrum of an OEM clear coat.

## 5. Conclusions

This study highlights the importance of applying the necessary data standardization and preprocessing methods to classify automotive clear coat spectra by vehicle manufacturer using neural networks. Because the FTIR spectra were obtained from different instruments, normalizing the helium–neon laser frequency to the same value for each instrument ensured that each IR spectrum was represented by the same number of points. Data preprocessing, specifically baseline correction, removal of sample outliers, and normalizing each IR spectrum to unit length, was necessary to identify fingerprint patterns in the mid-IR spectral data of the automotive clear coats characteristic of the motor vehicle manufacturer by the MLP models and to improve their generalization performance. Different wavenumber regions of the IR spectra were also investigated using these neural network models. The performance of each model was characterized by ten-fold cross-validation. Overfitting was not a problem in this study, as the prediction accuracy obtained with ten-fold cross-validation was comparable to the results obtained for the external prediction set by each of these models. The best results for these models were obtained when employing batch normalization and dropout with each hidden layer.

With the burgeoning demand for ownership of automotive vehicles, it will be necessary in a future study to increase the representation of some motor vehicle manufacturers in the dataset, e.g., Honda, and add other vehicle manufacturers to the training set, e.g., Hyundai, in response to market demand. Baseline correction is crucial for discriminating similar IR spectra. Although the rubber band algorithm reduced the amount of background noise in the IR spectra of automotive clear coats, the elimination of background from the IR spectra would further improve the performance of the MLP models developed in this study to discriminate similar IR spectra. An adaptive approach to baseline correction, recently developed in our laboratory [40], where the local baseline is used to estimate the baseline of subsequent spectra, would probably yield better results for the automotive clear coats. Outlier removal is also crucial, as discordant samples can prevent the network from generalizing. Confounding the detection of outliers using the generalized distance test in the vehicle manufacturer dataset is the clustering of the samples in each class due to differences in the polymer formulation used to prepare the automotive clear coats (e.g., acrylic melamine styrene versus acrylic melamine styrene polyurethane). An examination of the training data in smaller batches of samples using Q-Q plots [41] may do a better job at identifying discordant observations.

## Figures and Tables

**Table 1:** Automotive paint dataset.

Manufacturer	Number of Assembly Plants	Number of Spectra
General Motors	33	500
Chrysler	18	500
Ford	18	498
Honda	9	384
Nissan	7	414
Toyota	15	500
Total	100	2796

**Table 2:** Training set.

Manufacturer	Number of Spectra
General Motors	400
Chrysler	400
Ford	398
Honda	307
Nissan	331
Toyota	401
Total	2237

**Table 3:** Prediction set.

Manufacturer	Number of Spectra
General Motors	100
Chrysler	100
Ford	100
Honda	77
Nissan	83
Toyota	99
Total	559

**Table 4:** Sample outliers identified by the generalized distance test.

	4000–376 cm^−1^	1500–600 cm^−1^	1844–667 cm^−1^	1641–667 cm^−1^	1641–860 cm^−1^
GM	2	2	2	4	5
Chrys	18	5	17	8	8
Ford	3	0	9	1	2
Honda	3	3	3	3	0
Nissan	14	0	15	2	1
Toyota	16	17	17	2	2
Total	56	27	63	20	18

**Table 5:** Summary of results for the full spectral range models.

Model	Epochs	Training Set	Prediction Accuracy
CNN3ANN4	50,000	100%	87.5%
ANN4	50,000	100%	88.2%
ANN4	5000	100%	86.5%
ANN4	500	100%	81.7%

**Table 6:** Summary of modeling results for different spectral ranges.

Model	Epochs	Training Set	Prediction Accuracy
ANN4 (4000–376 cm^−1^)	80,000	100%	87.9%
ANN4 (1500–600 cm^−1^)	80,000	100%	89.45%
ANN4 (1844–667 cm^−1^)	80,000	99.999%	90.06%
ANN4 (1641–667 cm^−1^)	80,000	100%	89.98%
ANN4 (1641–860 cm^−1^)	80,000	100%	88.23%

**Table 7:** Confusion matrix for full spectral range model.

	GM	Chrysler	Ford	Honda	Nissan	Toyota
GM	84.2%	2.5%	6.8%	2%	3.2%	1.3%
Chrysler	2.4%	87.7%	6.8%	0%	1.8%	1.3%
Ford	9.6%	5.6%	81%	1.8%	0%	2%
Honda	1%	0	0.7%	93.8%	3.5%	1%
Nissan	3.2%	0	2.8%	2.2%	89.9%	1.9%
Toyota	2.1%	0.8%	0.7%	2.1%	1.3%	93%

**Table 8:** Accuracy of the models for the external test set.

Segment Used for Training	^1^ Prediction Accuracy	^2^ Accuracy	^3^ Precision	^4^ Recall	^5^ F1 Score
4000–376 cm^−1^	86.58%	95.53%	86.58%	86.58%	86.58%
1500–600 cm^−1^	87.29%	95.77%	87.30%	87.30%	87.30%
1844–667 cm^−1^	86.05%	95.35%	86.05%	86.05%	86.05%
1641–667 cm^−1^	88.37%	96.12%	88.37%	88.37%	88.37%
1641–860 cm^−1^	85.51%	95.17%	85.51%	85.51%	85.51%

^1^ $\frac{Number\;of\;Samples\;Correctly\;Classified}{Total\;Number\;of\;Samples}$, ^2^ $\frac{\sum TP+\sum TN}{TP+TN+\sum FP+\sum FN}\;where\;TP=true\;positives,\;TN=true\;negatives,\;FP=false\;positives,\;\;FN=false\;negative$, ^3^ $\frac{\sum TP}{\sum TP+\sum FP}\;where\;TP=true\;positives\;and\;FP=false\;positives$, ^4^ $\frac{\sum TP}{\sum TP+\sum FN}\;where\;TP=true\;positives\;and\;FN=false\;negatives$, ^5^ $2\times \left(\frac{(\mathrm{precision}\times \mathrm{recall})}{(\mathrm{precision}+\mathrm{recall})}\right)$.

**Table 9:** Confusion matrix for segment C.

	GM	Chrysler	Ford	Honda	Nissan	Toyota
GM	82%	2%	6%	3%	3%	4%
Chrysler	2%	88%	8%	0%	0%	2%
Ford	6%	5%	86%	1%	2%	0%
Honda	0%	0%	1.3%	89.6%	3.9%	5.2%
Nissan	0%	1.2%	1.2%	1.2%	86.8%	9.6%
Toyota	0%	0%	1%	1%	0%	98%

## Data Availability

Data was obtained from the RCMP Forensic Laboratory and is available upon request from Barry K. Lavine (barry.lavine@okstate.edu) with the permission of the RCMP.

## Abbreviations

The following abbreviations are used in this manuscript:

OEM	Original equipment manufacturer
PDQ	Paint data query
IR	Infrared
FTIR	Fourier transform infrared spectroscopy
ANN	Artificial neural network
CNN	Convolutional neural network
MLP	Multilayer perceptron

## References

[1] Streitberger H.-J., Dossel K.-F. (2008). Automotive Paint and Coatings.

[2] Badzoka J., Kappacher C., Lauß J., Huck C.W. (2025). Accelerating Microplastic Analysis with Machine Learning: Utilizing Multi-Layer Perceptron (MLP) for Classification of Hyperspectral Data. Microchem. J..

[3] Pradhan P., Guo S., Ryabchykov O., Popp J., Bocklitz T.W. (2020). Deep Learning a boon for Biophotonics. J. Biophotonics.

[4] Bona E., Marquetti I., Varaschim Link J., Figueiredo Makimori G.Y., da Costa Arca V., Guimaraes Lemes A.L., Garcia Ferreira J.M., dos Santos Scholz M.B., Valderrama P., Poppi R.J. (2017). Support Vector Machines in Tandem with Infrared Spectroscopy for Geographical Classification of Green Arabica Coffee. LWT-Food Sci. Technol..

[5] Szymanska E., Gerretzen J., Engel J., Geurts B., Blanchet L., Buydens L.M.C. (2015). Chemometrics and Qualitative Analysis Have a Vibrant Relationship. TrAC.

[6] Fink F., Emmerling F., Falkenhagen J. (2021). Identification and Classification of Technical Lignins by Means of Principle Component Analysis and K-Nearest Neighbor Algorithm. Chem. Methods.

[7] Acquarelli J., van Laarhoven T., Gerretzen J., Tran T.N., Buydens L.M., Marchiori C.E. (2017). Convolution Neural Networks for Vibrational Spectroscopic Data Analysis. Anal. Chim. Acta.

[8] Duarte J.M., Silva Sales N.G., Sousa M.H., Bridge C., Maric M., de Andrade Gomes J. (2020). Automotive Paint Analysis: How Far Has Science Advanced in the Last Ten Years. TrAC.

[9] Fasasi A., Mirjankar N., Stoian R.-I., White C.G., Allen M., Sandercock M.P., Lavine B.K. (2015). Pattern Recognition Assisted Infrared Library Searching of Automotive Clear Coats. Appl. Spectros..

[10] Lavine B.K., Fasasi A., Mirjankar N., White C., Sandercock M. (2015). Search Prefilters to Assist in Library Searching of Infrared Spectra of Automotive Clear Coats. Talanta.

[11] Lavine B.K., Mirjankar N., Ryland S., Sandercock M. (2011). Wavelets and Genetic Algorithms Applied to Search Prefilters for Spectral Library Matching in Forensics. Talanta.

[12] Walker J.S. (1999). Primer on Wavelets and Their Scientific Applications.

[13] Lavine B.K., White C.G., Davidson C.E., Brown S.D., Tauler R., Walczak B. (2020). Genetic Algorithms for Variable Selection and Pattern Recognition. Comprehensive Chemometrics.

[14] Krohn J., Beyleveld G., Bassens A. (2020). Deep Learning Illustrate.

[15] Goodfellow I., Bengio Y., Courville A. (2016). Deep Learning.

[16] Crain Communications (2015). Automotive News.

[17] Rodgers P.G., Cameron R., Cartwright N.S., Clark W.H., Deak J.S., Norman E.W. (1976). The Classification of Automotive Paint by Diamond Window Infrared Spectrophotometry Part I: Binders and Pigments. Can. Soc. Forens. Sci. J..

[18] Rodgers P.G., Cameron R., Cartwright N.S., Clark W.H., Deak J.S., Norman E.W. (1976). The Classification of Automotive Paint by Diamond Window Infrared Spectrophotometry Part II: Automotive Topcoats and Undercoats. Can. Soc. Forens. Sci. J..

[19] Griffiths P.R., deHaseth J.A. (1986). Fourier Transform Infrared Spectroscopy.

[20] Shen X., Ye S., Xu L., Hu R., Jin L., Xu H., Liu J., Liu W. (2018). Study on Baseline Correction Methods for the Fourier Transform Infrared Spectra with Different Signal-to-Noise Ratios. Appl. Optics.

[21] Schwager S.J., Margolin B.H. (1982). Detection of Multivariate Normal Outliers. Ann. Stat..

[22] Stapanian M.A., Garner F.C., Fitzgerald K.E., Flatman G.T., Nocerino J.M.J. (1993). Finding suspected causes of measurement error in multivariate environmental data. J. Chemometrics.

[23] Scout 2008. https://www.epa.gov/land-research/proucl-software.

[24] Nair V., Hinton G. Rectified Linear Units Improved Restricted Boltzmann Machines. Proceedings of the International Conference on Machine Learning.

[25] Srivastava N., Hinton G., Krizhevsky A., Sutskever I., Salakhutdinov R. (2014). Dropout: A Simple Way to Prevent Neural Networks from Overfitting. J. Mach. Learn. Res..

[26] Ioffe S., Szegedy C. (2015). Batch Normalization: Accelerating Deep Network Training by Reducing Internal Covariate Shift. Int. Conf. Mach. Learn..

[27] Bridle J.S., Soulié F.F., Hérault J. (1990). Probabilistic Interpretation of Feedforward Classification Network Outputs, with Relationships to Statistical Pattern Recognition. Neurocomputing. NATO ASI Series.

[28] Rumelhart David E., Hinton Geoffrey E., Williams Ronald J. (1986). Learning Representations by Back-Propagating Errors. Nature.

[29] Kingma D., Ba J. Adam: A Method for Stochastic Optimization. Proceedings of the 3rd International Conference on Learning Representations.

[30] Murphy K. (2022). Probabilistic Machine Learning: An Introduction.

[31] Affadu-Danful G.P., Zhong H., Sharma Dahal K., Kalkan K., Zhang L., Lavine B.K. (2023). Raman Spectroscopy to Enhance Investigative Lead Information in Automotive Clearcoats. Appl. Spectrosc..

[32] Yang J., Xu J., Zhang X., Wu C., Lin T., Ying Y. (2019). Deep Learning for Vibrational Spectroscopic Analysis: Recent Progress and a Practical Guide. Anal. Chim. Acta.

[33] Booksh K.S., Lavine B.K., Neal S.L. (2024). The Future of Molecular-Scale Measurements Enabled by Chemical Data Science: A Report on Envisioning Data Driven Advances in Measurements, An NSF-Funded Workshop. Appl. Spectros. Pract..

[34] Frank I.E., Friedman J.H. (1993). A Statististical View of Some Chemometrics Regression Tools. Technometrics.

[35] Brown C.D. (2004). Discordance Between Net Analyte Signal Theory and Practical Multivariate Calibration. Anal. Chem..

[36] Abrahamsson C., Johansson J., Sparen A., Lindgren F. (2003). Comparison of Different Variable Selection Methods Conducted on NIR Transmission Measurements on Intact Tablets. Chemom. Intell. Lab. Systems.

[37] Balabin R.M., Smirnov S.V. (2011). Variable Selection in Near-Infrared Spectroscopy: Benchmarking of Feature Selection Methods on Biodiesel Data. Anal. Chim. Acta.

[38] Guyon I., Gunn S., Nikravesh M., Zadeh L. (2006). Feature Extraction: Foundations and Applications.

[39] Lavine B.K., White C.G., Ding T. (2018). Library Search Prefilters for Vehicle Manufacturer to Assist in the Forensic Examination of Automotive Paints. Appl. Spectros..

[40] Zhong H. (2024). Ultramicrotomy and Infrared Imaging Applied to the Forensic Examination of Automotive Paint. Ph.D. Dissertation.

[41] Johnson R.A., Wichern D.W. (1982). Applied Multivariate Statistical Analysis.

